# An adult with cystathionine beta-synthase deficiency, camptodactyly-arthropathy-coxa vara-pericarditis syndrome, and deafness: A case report

**DOI:** 10.1590/1678-4685-GMB-2022-0335

**Published:** 2024-04-08

**Authors:** Karina Carvalho Donis, Marco Antônio Baptista Kalil, Fabiano Poswar, Fernando Kok, Charles Lubianca Kohem, Soraia Poloni, Taciane Borsatto, Filippo Pinto e Vairo, Franciele Cabral Pinheiro, Ida Vanessa Doederlein Schwartz

**Affiliations:** 1 Universidade Federal do Rio Grande do Sul, Programa de Pós-Graduação em Genética e Biologia Molecular, Porto Alegre, RS, Brazil.; 2 Universidade Federal do Rio Grande do Sul, Hospital de Clínicas de Porto Alegre, Serviço de Genética Médica, Porto Alegre, RS, Brazil.; 3 Universidade Federal de Ciências da Saúde de Porto Alegre, Porto Alegre, RS, Brazil.; 4 Universidade de São Paulo, Hospital das Clínicas, Departamento de Neurologia, Unidade de Neurogenética, São Paulo, SP, Brazil.; 5 Mendelics Análise Genômica, São Paulo, SP, Brazil.; 6 Universidade Federal do Rio Grande do Sul, Hospital de Clínicas de Porto Alegre, Serviço de Reumatologia, Porto Alegre, RS, Brazil.; 7 Universidade Federal do Rio Grande do Sul, Hospital de Clínicas de Porto Alegre, Centro de Pesquisa Experimental, Laboratório BRAIN, Porto Alegre, RS, Brazil.; 8 Mayo Clinic, Center for Individualized Medicine, Rochester, MN, USA.; 9 Mayo Clinic, Department of Clinical Genomics, Rochester, MN, USA.

**Keywords:** Classic Homocystinuria, Camptodactyly-Arthropathy-Coxa Vara-Pericarditis Syndrome, deafness, exome analysis, multilocus pathogenic variation

## Abstract

Massive sequencing platforms allow the identification of complex clinical phenotypes involving more than one autosomal recessive disorder. In this study, we report on an adult patient, born to a related couple (third degree cousins), referred for genetic evaluation due to *ectopia lentis,* deafness and previous diagnosis of juvenile idiopathic arthritis. He was biochemically diagnosed as having Classic Homocystinuria (HCU); Sanger sequencing of the *CBS* gene showed the genotype NM_000071.2(*CBS*):c.[833T>C];[833T>C], compatible with the diagnosis of pyridoxine-responsive HCU. As he also had symptoms not usually associated with HCU, exome sequencing was performed. In addition to the variants found in the Sanger sequencing, the following variants were identified: NM_001256317.1(*TMPRSS3*):c.[413C>A];[413C>A]; and the NM_005807.6(*PRG4*):c.[3756dup]:[3756dup], confirming the diagnosis of autosomal recessive nonsyndromic deafness and Camptodactyly-Arthropathy-Coxa Vara-Pericarditis Syndrome (CACP), respectively. Genomic analysis allowed the refinement of the diagnosis of a complex case and improvement of the patient’s treatment.

The concept of multilocus pathogenic variations (MPV) is relatively new, corresponding to the occurrence of two or more monogenic disorders in the same individual, which leads to complex phenotypes difficult to identify only by clinical or biochemical tests (Baltaci et al., 2021; Correia-Costa et al., 2022; Herman et al., 2022). Since the advent of genome/exome sequencing, MPVs have been identified in 1.4% to 7.2% of patients with rare diseases (Yang et al., 2013, 2014; Posey et al., 2017; Correia-Costa *et al.*, 2022). For autosomal recessive disorders, homozygous MPVs can reach up to 29% of patients in populations that present high rates of consanguineous marriage (Mitani et al., 2021). 

We report herein an unprecedented case of an adult patient with *ectopia lentis*, juvenile idiopathic arthritis (JIA), deafness, and psychiatric disorder who ended up being diagnosed with three monogenic diseases. This study was approved by the Local Research Ethics Committee and the patient provided written informed consent to the analyses and publication of the results. 

A 33-year-old male patient was sent to genetic evaluation due to multisystemic symptoms. At physical exam, he presented weight at p90, height between p50-90, no facial dysmorphisms, contractures in hands (camptodactyly) and elbows, and genu valgum. He was the first-born of a related couple (the parents were third cousins), with no family history of genetic diseases (Figure 1). 

**Figure 1 -:**
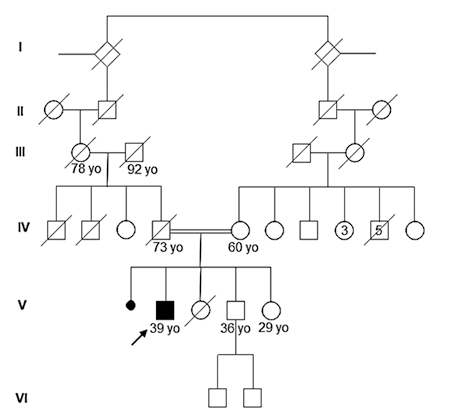
Family History. The family pedigree representing six generations. Subject IV-4 died of acute myeloid leukemia. Subject V-2 died at 5 months (skin lesions/meningitis/hospitalized for 3 months). No other family member presents clinical manifestations of Classic Homocystinuria or Camptodactyly-Arthropathy-Coxa Vara-Pericarditis Syndrome or autosomal recessive nonsyndromic deafness.

At the age of three months, he presented contractures in his hands, being submitted to surgical correction at one year of age. At the age of two, he presented edema in his knees and at six, he was evaluated by a rheumatologist and diagnosed with JIA. At that time, inflammatory markers were normal and treatment with methotrexate and corticosteroids was started. His psychomotor development was normal, and he developed bilateral hearing loss at age 9. He had right and left total hip arthroplasty at the age of 20 and 21, respectively, considered at that time to be secondary to the prolonged corticosteroid treatment; surgery for *ectopia lentis* at 23 years of age; and a single seizure at age 26. No acetabular cyst was detected (Figure 2). Echocardiogram performed at age 29 showed mild aortic insufficiency. After a few years, he started having auditory hallucinations, but no definitive psychiatric diagnosis was made. A hand X-ray was performed at age 37 and showed periarticular osteopenia, deformity with subluxation of the bilateral proximal interphalangeal joints, bone proliferations along the bilateral ulnar radio articulations, pseudocysts in the styloid processes, and tenuous images with calcic attenuation projected laterally in the region of the triangular fibrous cartilage (Figure 3).

**Figure 2 -:**
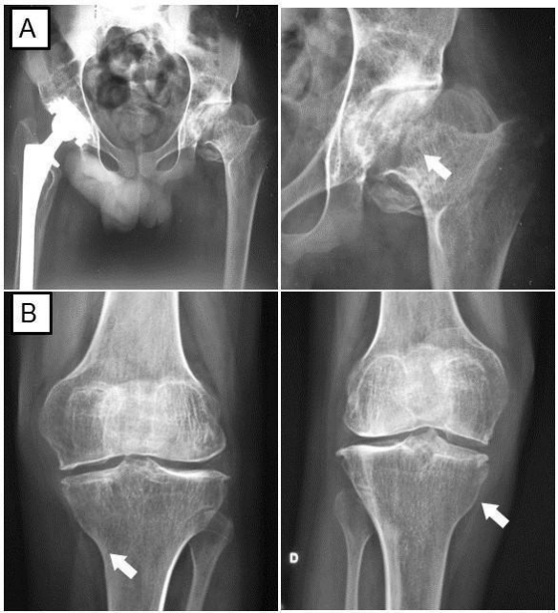
Bilateral hip and knee X- ray showing osteonecrosis of the left femur head and right prosthesis (A) and osteopenia (B).

**Figure 3 -:**
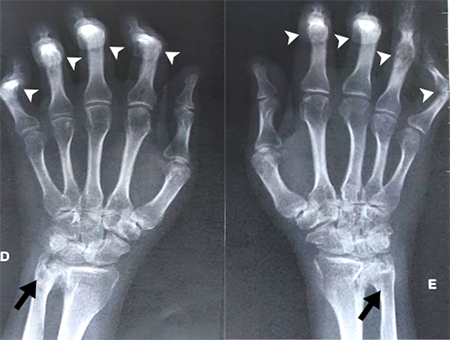
Bilateral hand X- ray. Pseudocysts are seen in the styloid processes (arrows) and bilateral deformity with subluxation of proximal interphalangeal joints (arrowheads).

A comprehensive biochemical investigation was performed and showed serum total homocysteine (tHcy) 431 umol/L (Reference range: 5-15) and methionine: 42 umol/L (Ref: 13-37). Due to a presumptive diagnosis of classical homocystinuria (HCU, OMIM 236200) diagnosis, treatment with pyridoxine 500 mg/day was initiated and the tHcy level decreased to 31 umol/L. 

Targeted genetic analysis (Sanger sequencing of the *CBS* gene) confirmed a homozygous pathogenic variant in *CBS* NM_000071.2:c.[833T>C]:[833T>C] (p.(Ile278Thr)), which is located at exon 8, and associated with pyridoxine responsiveness (Figure 4a ). However, HCU alone did not explain the JIA diagnosis; hand and elbows contractures; and hearing loss. Thus, exome sequencing was performed (exon capture with Nextera Exome Capture followed by massive parallel sequencing with Illumina HiSeq, using the GRCh37 version of the human genome as reference). In addition to the pathogenic variant in *CBS*, the exome sequencing revealed a homozygous pathogenic variant in *TMPRSS3* NM_001256317.1:c.[413C>A]:[413C>A] (p.(Ala138Glu)), which is located at exon 5, that has been associated with nonsyndromic autosomal recessive deafness (OMIM 605511), as well as a homozygous likely pathogenic variant in *PRG4* NM_005807.6:c.[3756dup]:[3756dup] (p.(Lys1253Ter)), which is located at exon 10, related to Camptodactyly-Arthropathy-Coxa Vara-Pericarditis (CACP) Syndrome (OMIM 208250; Figure 4b  -d). At the time all diagnoses were made, his medication regimen was: pyridoxine 500 mg/day, folic acid 5 mg/day, valproic acid 750 mg 3 times/day, olanzapine 15 mg/day, phenobarbital 100 mg/day, escitalopram 10 mg/day, and methotrexate 5 mg twice a month. Once the JIA diagnosis was dismissed, methotrexate was stopped. All his siblings were evaluated biochemically and clinically, and had normal levels of tHcy and methionine, normal hearing, and normal musculoskeletal exam. A limitation of this study is that the parents and siblings of the proband were not genetically investigated to confirm or exclude the carrier status. Genetic counseling was provided to the family.

**Figure 4 -:**
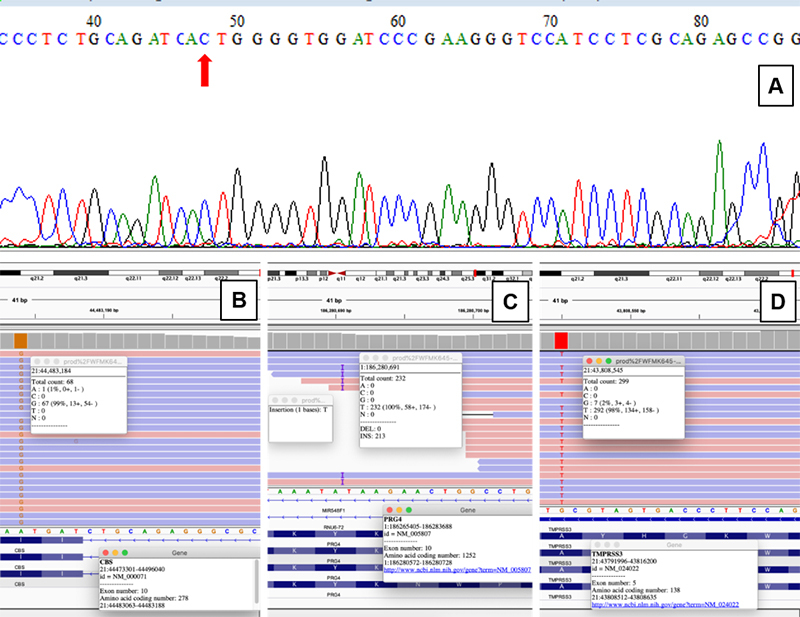
Genetic analyses of *CBS, PRG4* and *TMPRSS3* genes. A) electropherogram of patient showing the pathogenic variant NM_000071.2(CBS):c.833T>C (p.Ile278Thr) in homozygosis in *CBS* gene. The red arrow indicates the position of variant. Below, pathogenic variants identified through exome sequencing in *CBS* (B), *PRG4* (C), and *TMPRSS3* (D) genes.

Exome sequencing may be used if, despite consistent phenotypic features, a single genetic locus cannot explain the full phenotypic spectrum of a condition (Karaca et al. 2018). However, the large amount of data generated requires a careful analysis to detect significant variants for patient’s disorders. In this sense, a detailed description of the patient’s clinical manifestations is essential for the correct diagnosis. Data suggest exome sequencing results in diagnoses of 30-50% of patients tested and around 4.6-7% of patients are diagnosed with two independent monogenic conditions (Posey et al., 2017; Yang et al., 2013, 2014; Ferrer et al., 2019).

After the genetic evaluation, the first clinical hypothesis and diagnosis in this patient was HCU, with a pyridoxine-responsive phenotype (Shih et al., 1995). HCU is a rare autosomal recessive inborn error of metabolism, due to impaired conversion of Hcy to cystathionine leading to hyperhomocysteinemia. There is a wide range of clinical manifestations, varying from severe childhood-onset multisystem disease to asymptomatic individuals until adulthood. Main findings are *ectopia lentis*, osteoporosis, ‘marfanoid’ habitus, learning difficulties, psychiatric disorders and predisposition to thromboembolism (Morris et al., 2017).

HCU is typically classified into three phenotypes: pyridoxine-responsive, partial pyridoxine-responsive, and pyridoxine-unresponsive. Individuals with the responsive form usually have milder phenotypes and are treated with pyridoxine (vitamin B6) and folic acid. On the other hand, unresponsive patients may require, in addition to pyridoxine and folic acid, a methionine restricted diet with methionine-free formula supplementation, and betaine. Most of the individuals with atypical or attenuated phenotypes remain undiagnosed for several years (Morris et al., 2017). The treatment prevents the occurrence of thromboembolism, which is the main cause of death of these patients. The p.(Ile278Thr) variant in the *CBS* gene has been described as the most widely dispersed variant, and homozygous patients present a mild phenotype, usually responsive to pyridoxine treatment (Weber Hoss et al., 2020).

In addition, the patient had some atypical findings such as hearing loss and JIA, that could not be explained by HCU. After performing exome sequencing to clarify the phenotype, he was diagnosed with two other autosomal recessive diseases: CACP syndrome and recessive nonsyndromic deafness. Furthermore, the variant in the *TMPRSS3* gene is just 675 Kb from the variant present in the *CBS* gene, on chromosome 21, so both are likely to be segregated in conjunction. Thus, it is important to point out the occurrence of parental consanguinity as a risk factor for the autosomal recessive diseases in the offspring.

Consanguinity means that two people are descended from the same ancestor. In practice, the likelihood of sharing carrier status for a rare autosomal recessive disease is greater for related couples than for unrelated individuals. However, this increased risk does not apply to offspring if the common ancestor of the couple is more remote than second-degree cousins (Fareed and Afzal, 2017). On the other hand, it has been estimated that every individual carries more than 20 pathogenic variants associated to autosomal recessive diseases, but these estimates may vary in different populations considering aspects such as founder effect, endogamy, non-random mating among others. For example, in Northern Europe the estimated minimum risk of an unrelated couple having a child with an autosomal recessive disorder is 1 in 628 pregnancies (Antonarakis, 2019). In the case presented herein, the parents referred to be third cousins. Therefore, the theoretical probability of the couple having a child with at least one autosomal recessive disorder is the same as that presented by the general population. However, the patient is homozygous for all the rare variants found (autosomal recessive patients born to non-related couples are usually compound heterozygotes, unless the variant is relatively frequent), which reinforces the importance of the consanguinity in this case. Besides that, the haplotype including pathogenic variants in *CBS* and *TMPRSS3* genes can be explained by the relationship between his parents. As the history of the family was obtained by interview, we cannot exclude a mistake on the reported consanguinity, although the occurrence of three monogenic diseases in the same individual can also be random or explained by other factors. For example, since the variants found have not been searched in both parents, we cannot exclude non-maternity and/or non-paternity.

Pathogenic variants in *TMPRSS3* have been implicated in prelingual and postlingual hearing impairment. There is a genotype-phenotype correlation where loss-of-function variants cause the most severe phenotype, while missense variants are associated with a milder form of hearing impairment (Gao et al., 2017). The p.(Ala138Glu) variant has been associated with severe-to-profound hereditary high-frequency sensorineural hearing loss before 15 years of age. Furthermore, patients carrying this variant have poor cochlear implant performance, which is the gold-standard treatment for this disorder (Chen et al., 2022). Our patient had postlingual hearing impairment at nine years such as described in patients from other studies (Weegerink et al., 2011). 

Proteoglycan 4 (PRG4) is expressed in chondrocytes of the superficial zone and is involved in lubricating and protecting cells in the surfaces of joints and tendons as well as in non-skeletal tissue including liver and pericardium (Albuhairan and Al-Mayouf, 2013). Pathogenic variants in *PRG4* identified on chromosome 1q25-q31 cause the CACP syndrome, characterized by congenital or early-onset camptodactyly, childhood-onset non-inflammatory arthropathy of large joints - such as elbows, hips, knees, and ankles - synovial hyperplasia, progressive coxa vara deformity and non-inflammatory pericardial or pleural effusion (Albuhairan and Al-Mayouf, 2013; Vutukuru and Reddy, 2016). Due to overlapping symptoms, CACP may be misdiagnosed as JIA, which is treated with immunomodulators (Kisla Ekinci et al., 2021). Importantly, the specific musculoskeletal features of CACP syndrome do not respond to this type of medication, therefore affected individuals should be treated with pain medications and physical therapy (Albuhairan and Al-Mayouf, 2013). Some clinical features that may help to differentiate CACP from JIA are the presence of coxa vara, positive family history, normal inflammatory markers, absence of inflammation in synovial aspirates or synovial biopsies, and typical imaging findings including the presence of intraosseous cysts (Marcelino et al., 1999; Madhusudan et al., 2016).

Providing patient with the correct diagnosis allowed us to withdraw methotrexate which is known to cause serious and long-term severe adverse effects such as predisposition to infections, lung and skin problems, dizziness, and gastrointestinal issues as well as tailor the physical therapy and other medications to treat his diseases. Besides that, methotrexate can increase the tHcy levels.

Here, we report the success of exome sequencing in diagnosing an individual with three autosomal recessive disorders. This case highlights the importance of further investigations when patient’s symptoms are not explained by a single disease. An accurate diagnosis of the coexistence of multiple conditions can allow for the cessation of ineffective and potentially harmful treatments.

## References

[B1] Albuhairan I, Al-Mayouf SM (2013). Camptodactyly-arthropathy-coxavara-pericarditis syndrome in Saudi Arabia: Clinical and molecular genetic findings in 22 patients. Semin Arthritis Rheum.

[B2] Antonarakis SE (2019). Carrier screening for recessive disorders. Nat Rev Genet.

[B3] Baltaci HNC, Taşdelen E, Topçu V, Eminoǧlu FT, Karabulut HG (2021). Dual diagnosis of Ochoa syndrome and Niemann-Pick disease type B in a consanguineous family. J Pediatr Endocrinol Metab.

[B4] Chen YS, Cabrera E, Tucker BJ, Shin TJ, Moawad JV, Totten DJ, Booth KT, Nelson RF (2022). TMPRSS3 expression is limited in spiral ganglion neurons: Implication for successful cochlear implantation. J Med Genet.

[B5] Correia-Costa GR, Santos AM, Leeuw N, Rigatto SZP, Belangero VMS, Steiner CE, Gil-da-Silva-Lopes VL, Vieira TP (2022). Dual molecular diagnoses of recessive disorders in a child from consanguineous parents: Case report and literature review. Genes.

[B6] Kisla Ekinci RM, Balci S, Dogan H, Ceylaner S, Varan C, Erdem S, Coban F, Bisgin A (2021). Camptodactyly-arthropathy-coxa vara-pericarditis syndrome resembling juvenile idiopathic arthritis: A single-center experience from Southern Turkey. Mol Syndromol.

[B7] Fareed M, Afzal M (2017). Genetics of consanguinity and inbreeding in health and disease. Ann Hum Biol.

[B8] Ferrer A, Schultz-Rogers L, Kaiwar C, Kemppainen JL, Klee EW, Gavrilova RH (2019). Three rare disease diagnoses in one patient through exome sequencing. Cold Spring Harbor Mol Case Stud.

[B9] Gao X, Huang SS, Yuan YY, Xu JC, Gu P, Bai D, Kang DY, Han MY, Wang GJ, Zhang MG (2017). Identification of TMPRSS3 as a significant contributor to autosomal recessive hearing loss in the Chinese population. Neural Plast.

[B10] Herman I, Jolly A, Du H, Dawood M, Abdel-Salam GMH, Marafi D, Mitani T, Calame DG, Coban-Akdemir Z, Fatih JM (2022). Quantitative dissection of multilocus pathogenic variation in an Egyptian infant with severe neurodevelopmental disorder resulting from multiple molecular diagnoses. Am J Med Genet A.

[B11] Karaca E, Posey JE, Akdemir ZC, Pehlivan D, Harel T, Jhangiani SN, Bayram Y, Song X, Bahrambeigi V, Yuregir OO (2018). Phenotypic expansion illuminates multilocus pathogenic variation. Genet Med.

[B12] Madhusudan S, Gupta A, Prakash M, Matta D, Suri D, Singh S (2016). Camptodactyly-arthropathy-coxa vara-pericarditis (CACP) syndrome: A mimicker of juvenile idiopathic arthritis. Scand J Rheumatol.

[B13] Marcelino J, Carpten JD, Suwairi WM, Gutierrez OM, Schwartz S, Robbins C, Sood R, Makalowska I, Baxevanis A, Johnstone B (1999). CACP, encoding a secreted proteoglycan, is mutated in camptodactyly-arthropathy-coxa vara-pericarditis syndrome. Nat Genet.

[B14] Mitani T, Isikay S, Gezdirici A, Gulec EY, Punetha J, Fatih JM, Herman I, Akay G, Du H, Calame DG (2021). High prevalence of multilocus pathogenic variation in neurodevelopmental disorders in the Turkish population. Am J Hum Genet.

[B15] Morris AAM, Kožich V, Santra S, Andria G, Ben-Omran TIM, Chakrapani AB, Crushell E, Henderson MJ, Hochuli M, Huemer M (2017). Guidelines for the diagnosis and management of cystathionine beta-synthase deficiency. J Inherit Metab Dis.

[B16] Posey JE, Harel T, Liu P, Rosenfeld JA, James RA, Akdemir ZHC, Walkiewicz M, Bi W, Xiao R, Ding Y (2017). Resolution of disease phenotypes resulting from multilocus genomic variation. N Engl J Med.

[B17] Shih VE, Fringer JM, Mandell R, Kraus JP, Berry GT, Heidenreich RA, Korson S, Levy HL, Ramesh V (1995). A missense mutation (I278T) in the cystathionine beta-synthase gene prevalent in pyridoxine-responsive homocystinuria and associated with mild clinical phenotype. Am J Hum Genet.

[B18] Vutukuru R, Reddy KKM (2016). Pathognomonic acetabular cysts in camptodactyly-arthropathy-coxa vara-pericarditis (CACP) syndrome. Indian J Med Res.

[B19] Weber Hoss GR, Sperb‐Ludwig F, Schwartz IVD, Blom HJ (2020). Classical homocystinuria: A common inborn error of metabolism? An epidemiological study based on genetic databases. Mol Genet Genomic Med.

[B20] Weegerink NJD, Schraders M, Oostrik J, Huygen PLM, Strom TM, Granneman S, Pennings RJE, Venselaar H, Hoefsloot LH, Elting M (2011). Genotype-phenotype correlation in DFNB8/10 families with TMPRSS3 mutations. J Assoc Res Otolaryngol.

[B21] Yang Y, Muzny DM, Reid JG, Bainbridge MN, Willis A, Ward PA, Braxton A, Beuten J, Xia F, Niu Z (2013). Clinical whole-exome sequencing for the diagnosis of mendelian disorders. N Engl J Med.

[B22] Yang Y, Muzny DM, Xia F, Niu Z, Person R, Ding Y, Ward P, Braxton A, Wang M, Buhay C (2014). Molecular findings among patients referred for clinical whole-exome sequencing. JAMA.

